# Do incentives, reminders or reduced burden improve healthcare professional response rates in postal questionnaires? two randomised controlled trials

**DOI:** 10.1186/1472-6963-12-250

**Published:** 2012-08-14

**Authors:** Liz Glidewell, Ruth Thomas, Graeme MacLennan, Debbie Bonetti, Marie Johnston, Martin P Eccles, Richard Edlin, Nigel B Pitts, Jan Clarkson, Nick Steen, Jeremy M Grimshaw

**Affiliations:** 1 Leeds Institute of Health Sciences, University of Leeds, Leeds, UK; 2 Health Services Research Unit, University of Aberdeen, Aberdeen, UK; 3 Dental Health Services Research Unit, University of Dundee, Dundee, UK; 4 College of Life Sciences and Medicine, University of Aberdeen, Aberdeen, UK; 5 Institute of Health and Society, Newcastle University, Newcastle upon Tyne, UK; 6 School of Population Health, University of Auckland, Auckland, New Zealand; 7 Clinical Epidemiology Programme, Ottawa Health Research Institute and Department of Medicine, University of Ottawa, Ottawa, Canada

## Abstract

**Background:**

Healthcare professional response rates to postal questionnaires are declining and this may threaten the validity and generalisability of their findings. Methods to improve response rates do incur costs (resources) and increase the cost of research projects. The aim of these randomised controlled trials (RCTs) was to assess whether 1) incentives, 2) type of reminder and/or 3) reduced response burden improve response rates; and to assess the cost implications of such additional effective interventions.

**Methods:**

Two RCTs were conducted. In RCT A general dental practitioners (dentists) in Scotland were randomised to receive either an incentive; an abridged questionnaire or a full length questionnaire. In RCT B non-responders to a postal questionnaire sent to general medical practitioners (GPs) in the UK were firstly randomised to receive a second full length questionnaire as a reminder or a postcard reminder. Continued non-responders from RCT B were then randomised within their first randomisation to receive a third full length or an abridged questionnaire reminder. The cost-effectiveness of interventions that effectively increased response rates was assessed as a secondary outcome.

**Results:**

There was no evidence that an incentive (52% versus 43%, Risk Difference (RD) -8.8 (95%CI −22.5, 4.8); or abridged questionnaire (46% versus 43%, RD −2.9 (95%CI −16.5, 10.7); statistically significantly improved dentist response rates compared to a full length questionnaire in RCT A. In RCT B there was no evidence that a full questionnaire reminder statistically significantly improved response rates compared to a postcard reminder (10.4% versus 7.3%, RD 3 (95%CI −0.1, 6.8). At a second reminder stage, GPs sent the abridged questionnaire responded more often (14.8% versus 7.2%, RD −7.7 (95%CI −12.8, -2.6). GPs who received a postcard reminder followed by an abridged questionnaire were most likely to respond (19.8% versus 6.3%, RD 8.1%, and 9.1% for full/postcard/full, three full or full/full/abridged questionnaire respectively). An abridged questionnaire containing fewer questions following a postcard reminder was the only cost-effective strategy for increasing the response rate (£15.99 per response).

**Conclusions:**

When expecting or facing a low response rate to postal questionnaires, researchers should carefully identify the most efficient way to boost their response rate. In these studies, an abridged questionnaire containing fewer questions following a postcard reminder was the only cost-effective strategy. An increase in response rates may be explained by a combination of the number and type of contacts. Increasing the sampling frame may be more cost-effective than interventions to prompt non-responders. However, this may not strengthen the validity and generalisability of the survey findings and affect the representativeness of the sample.

## Background

Postal questionnaires can provide a cost-effective method of surveying the views and opinions of a large number of participants in a literate population [1]. However, low response rates to postal questionnaires can introduce bias and subsequently threaten the validity of the study results. Postal questionnaires are commonly used in health care professional research [2,3], but response rates are declining [4]. Reviews of healthcare professional response rates [5,6] have found lower response rates than other respondent groups such as patients [7]. Studies included in these reviews commonly fail to use interventions proven to improve response rates in other populations (such as financial incentives and reduced response burden [3,8]). Methods to improve response rates do incur costs and increase the cost of research projects. There is a lack of evidence to inform the choice of intervention to improve healthcare professional response rates to postal questionnaires [4].

This paper reports two randomised controlled trials (RCTs) of three methods aiming to improve the response rate to two postal questionnaire surveys of health professionals. The two surveys (postal questionnaires) were designed to measure theory-based cognitions (developed to understand, predict and influence behaviour) and interim outcome measures (stated intention and behavioural simulation) for two clincial behaviours. These two surveys are part of a series of studies called the PRIME project (PRocess Modelling in Implementation Research [9-12]). PRIME aimed to develop a scientific rationale to design or choose an implementation intervention by identifying constructs from a range of psychological frameworks which would be predictive of evidence-based clinical behaviour. Full details of the methods and results of the PRIME studies are described elsewhere [9-12]. Following a lower than expected response rate (31%) to the radiograph postal questionnaire sent to general dental practitioners (dentists), the first in this series of studies [9], a decision was made to evaluate different methods to increase response rates in subsequent surveys. Interventions proven to increase response rates in other populations [3,8] were evaluated in two RCTs, designed to assess the effectiveness and efficiency of three interventions: an incentive; the type of reminder (questionnaire or postcard); and reduced response burden (an abridged questionnaire with less questions) to improve health care professional response rates to postal questionnaires.

## Methods

### Aim of these studies

To assess whether 1) an incentive, 2) type of reminder and/or 3) reduced response burden improve response rates; and to assess the cost implications of such additional effective interventions.

### Study design

**Figure 1:**
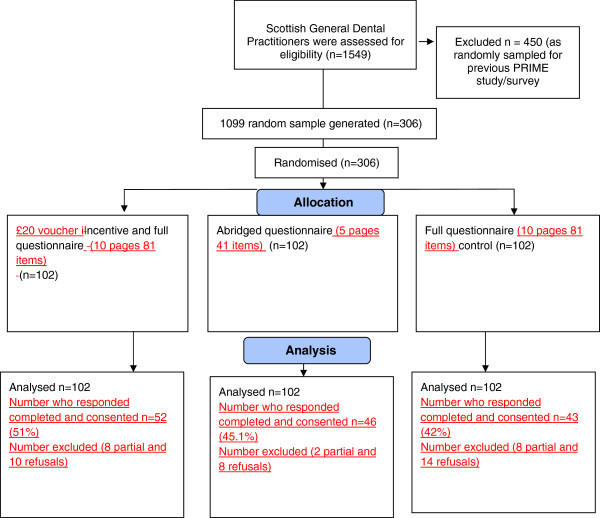
RCT A Trial flow diagram.

**Figure 2:**
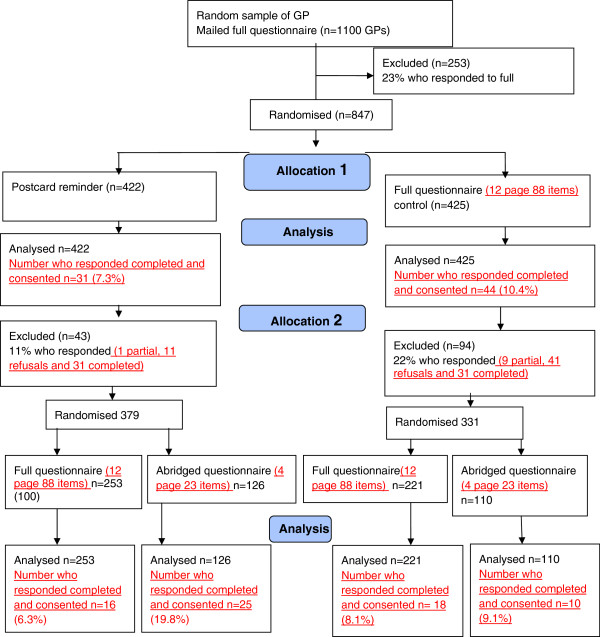
RCT B Trial flow diagram.

In RCT A a random sample of dentists in Scotland (who had not been invited to participate in the first PRIME study) were randomised to receive either an incentive; an abridged questionnaire or a full questionnaire (see Figure 1). In RCT B non-responders to a PRIME survey (a postal questionnaire sent to general medical practitioners (GPs) in the UK) were randomised, firstly to receive either a second copy of the full questionnaire or a postcard as a reminder. Continued GP non-responders were then randomised again stratified by their original allocated intervention to receive either a third copy of the questionnaire or abridged questionnaire (see Figure 2).

### Study population

#### RCT A

A simple random sample of general dental practitioners was taken from the list of all dentists who practice in Scotland and contribute data to the Scottish Dental Practice Board and Practitioner Services (MIDAS) information system. The list excluded those dentists who had been part of the random sample invited to complete the first PRIME questionnaire.

#### RCT B

All general practioners who did not respond to a postal questionnaire survey within two weeks of the posting out date. RCT B was opportunistic and nested in a PRIME study [10]. The survey was sent to a random sample of 1100 GPs who practice in the UK generated from a list of GPs who practice in Grampian, Tayside and Lothian. The GPs who did not respond to this initial questionnaire within two weeks of posting became the sample for RCT B.

### Primary outcome

We defined response rate as the proportion of the number of completed questionnaires with consent to access behavioural data returned to the study office out of the total number sent out.

### Secondary outcomes

The cost of effective methods to increase response rate was assessed.

### Control and intervention groups

#### RCT A

Dentists were allocated to one of three study arms: 1) Control: A ten page full length (81 questions) questionnaire (n = 102), 2) Incentive: The ten page questionnaire plus a £20 book voucher incentive (n = 102) or 3) Burden: An abridged five page questionnaire (41 questions) (n = 102) (see Figure 1). A £20 voucher was selected as an approximate pro rate for the time taken to complete the questionnaire. University regulations prevented us from mailing a cash incentive.

#### RCT B

GPs were allocated to one of two arms: 1) Control: A 12-page (88 questions) questionnaire (full length) as reminder 2) A postcard with a brief reminder message (but no questionnaire). Those who did not respond after two weeks were then randomised again (stratified within their original randomisation) to receive a reminder constituting either the 1) full questionnaire or 2) an abridged 4-page (23 questions) questionnaire.

### Sample size calculations

#### RCT A

We calculated that 306 participants (102 per arm) would be sufficient at 80% power to detect a difference in response rates from 28% in the control group to 50% in the intervention groups with a 2.5% significance level (conservative for multiple tests). The response rate in the control arm was based upon the response rate to the first full PRIME postal questionnaire [9]. The sample size aimed to contribute as much data as possible to the original PRIME study [9].

### RCT B

RCT B was an opportunistic trial which took place within an ongoing survey, the sample for that survey had already been generated, and an a priori power calculation was not possible. GPs were randomly allocated to receive a full questionnaire reminder or a postcard reminder in a 1:1 ratio. Those who did not respond after two weeks were then randomised to receive either the full questionnaire or an abridged 4-page (23 questions) questionnaire in a 2:1 ratio. An uneven randomisation was chosen in the second randomisation to contribute as much data as possible to the original PRIME study [10].

### Randomisation

A statistician blinded to participant identity generated a simple randomly-selected sample of 306 Scottish dentists and performed the randomisation for RCT A and RCT B.

### Procedure

In both RCT A and RCT B dentist and GP participants were mailed an invitation pack (letter of invitation, allocated intervention, a form requesting consent to allow the research team to access the respondent’s radiograph or prescribing data from centrally held databases, a study newsletter, and a reply paid envelope. Dentists were allocated to receive an invitation pack containing a full questionnaire, a full questionnaire with a £20 book voucher, or an abridged questionnaire. No further reminders were mailed to dentist participants. GPs who responded to the invitation pack did not receive further study materials. GP non-responders were randomised to receive a full questionnaire or a postcard reminder. Continued GP non-responders were randomised within their original allocation to receive a full or an abridged questionnaire reminder (see flowcharts 1 and 2 for full details). Secretaries recording receipt of RCTA and RCTB survey packs were blinded to group assignment.

### Statistical analyses

The analysis was performed on an intention-to-treat basis. In RCT A comparisons were made between the control and abridged group, and the control and incentive group for the primary outcome using logistic regression. In RCT B comparisons were made between the control and postcard reminder, and then by the level of burden for the primary outcome using logistic regression. The absolute percentage difference and 95% confidence interval derived from the logistic regression models are presented. All analyses were carried out using Stata (StataCorp. 2009. *Stata Statistical Software: Release 11*. College Station, TX: StataCorp LP.) [13].

### Cost-effectiveness analyses

Following the effectiveness analysis the costs of producing and posting each intervention in RCT B (i.e. postcard, full 12-page questionnaire, abridged 4-page questionnaire) and data entry were estimated at 2011 prices. RCT B data was used to define a series of different options by attributing any response to whichever intervention is current. For example, a GP who received a full questionnaire three times and responded to it the third time is assumed to have responded because of the third questionnaire, even though they have received the same materials twice before and may have responded anyway. This assumption allows a series of options to be identified that include stopping reminders at any stage. For example, this allows us to compare the cost-effectiveness of a single full questionnaire (using response rates in the study that identified our 847 participants) against a postcard reminder plus a second full questionnaire (see Figure 2).

A total of seven options were identified in this way and each is identified using a three digit code indicating the materials used at each stage (Q = 12 page questionnaire, q = 4 page questionnaire, p = postcard, x = none). For example, Qpq would refer to an initial full questionnaire followed by a postcard reminder and an abridged questionnaire, whilst Qxx would be just the initial full questionnaire with no further reminders. Any response (whether consenting or non-consent) would mean that no further intervention would be taken.

The seven options considered here are as follows: Qxx, Qpx Qpq, QpQ, QQx, QQq, QQQ. Each option was converted into a decision tree in TreeAge Pro 2011 [14], with all conditional probabilities modelled as Beta distributions using RCT B data. These Beta distributions allow us to reflect the fact that we can be more certain about response rates for the earlier interventions because more people received them. By drawing from these Beta distributions 50,000 times and running the model as Monte Carlo simulation, we can identify expected costs and response rates for each method and investigate which of these methods are likely to be the most efficient ways to increase response rates.

### Ethics statement

The original Process Modelling in Implementation Research (PRIME) questionnaires and an amendment to conduct the response rate trials were given favourable review by the UK South East Multi-Centre Research Ethics Committee (MREC/03/01/03).

## Results

Figures 1 and 2 outline the participant flow within RCT A and RCT B.

### RCT A

There was no statistically significant difference in the number of completed questionnaires between the groups sent a full (42.2%) or abridged (45.1%) questionnaire RD −2.9 (95%CI −16.5 to 10.7); (see Table 1). Sending a book voucher with the questionnaire increased the response rate (by almost 9% Incentive 51% versus full questionnaire 42.2%)) however this was not a statistically significant difference RD −8.8 (95%CI −22.5 to 4.8) (see Table 1).

**Table 1:** Response to reminders in RCT A

	**Full questionnaire (control)**		**Incentive**		**Abridged questionnaire**	
	**N = 102**		**N = 102**		**N = 102**	
	**n**	**%**	**n**	**%**	**n**	**%**
Completed questionnaires returned	43	42.2	52	51.0	46	45.1
Full questionnaire versus Incentive						
Absolute percent difference (95% CI) ; p-value	-8.8 ( -22.5 to 4.8);.21					
Full questionnaire versus abridged questionnaire						
Absolute percent difference (95% CI)	-2.9 (-16.5, 10.7);.67					
p value						

Percentage difference and 95% confidence intervals were derived from the logistic regression model, a positive difference favours the full questionnaire.

### RCT B

There was no evidence that a full questionnaire reminder improved response rates compared to a postcard reminder, 10.4% versus 7.3%, RD 3 (95%CI −0.1 to 6.8) (see Table 2). The abridged questionnaire sent to the continued non-responders achieved a statistically significant higher response rate than the full questionnaire, 14.8% versus 7.2%, RD 7.7 (95%CI 2.6 to 12.8) (see Table 3).

**Table 2:** Response to first reminder in RCT B

	**Full questionnaire**		**Postcard**	
	**N = 425**		**N = 422**	
	n	%	n	%
Completed questionnaires returned	44	10.4	31	7.3
Absolute percent difference (95% CI) ; p-value	3 (−0.1 , 6.8); 0.12			

**Table 3:** Response to second reminder in RCT B

	**Full questionnaire**		**Abridged questionnaire**	
	**N = 474**		**N = 236**	
	n	%	n	%
Completed questionnaires returned	34	7.2	35	14.8
Absolute percent difference (95% CI) ; p-value	-7.7 (−12.8 , -2.6); 0.001			

Continued non-responders were exposed to four intervention strategies that combined type of reminder with level of burden (see Figure 2). Results from our logistic regression comparing intervention strategies to three full questionnaires (response rate 8.1%) found that participants who received a postcard reminder followed by an abridged questionnaire were more likely to respond (19.8% RD 11.7 (95%CI 3.9 to 19.5); there was no evidence that those sent a postcard reminder followed by a full questionnaire or those sent a full questionnaire reminder followed by an abridged questionnaire had a different response rate when compared to those who were sent three full questionnaires (see Table 4).

**Table 4:** Response to second reminder in RCT B by first reminder received

	**Full questionnaire**				**Postcard**			
	**Full questionnaire**		**Abridged questionnaire**		**Full questionnaire**		**Abridged questionnaire**	
	**N = 221**		**N = 110**		**N = 253**		**N = 126**	
	n	%	n	%	n	%	n	%
Completed questionnaires returned	18	8.1	10	9.1	16	6.3	25	19.8
Absolute percentage difference (95% CI) compared to full questionnaire/full questionnaire combination; p-value								
Full/Abridged	-1.0 (− 7.6, 5.5); 0.77							
Postcard/Full	1.8 (−2.9, 6.5); 0.45							
Postcard/Abridged	-11.7 (−19.5, -3.9); 0.002							
Logistic regression model odds ratios (95% CI); p-value								
Abridged	1.13 (0.50, 2.53); 0.77							
Postcard	0.76 (0.38, 1.53); 0.45							
Interaction (Abridged * Postcard)	3.25 (1.13, 9.29); 0.028							

### Cost-effectiveness

The costs of materials in RCT B were estimated at £3.03 and £2.28 regardless of response for each 12-page and 4-page questionnaire, with data entry costs (for responses only) estimated at £2.54 and £1.27, respectively; postcard reminders were costed at £1.56. The total costs and expected response rates for the seven options are given in Table 5. If the aim is to increase the number of responses to a set figure, then the most cost-effective way of increasing the sample size would have been to increase the size of the initial sample beyond 1100 people (i.e. Qxx), since this provides additional respondents more cheaply (£15.71 per response) than any other option. Where this isn’t possible, or where other factors may be important, then following the sample up with a postcard and abridged questionnaire (Qpq) appears to be the next most cost-effective option with a marginally higher cost per additional response (£15.99).

**Table 5:** Response rates and Costs by option

	**Response rate**	**Average costs**	**Incr RR**	**Incr costs**	**Cost per additional response**
Qxx	0.23	£ 3.61	0.23	£ 3.61	£15.71 per response
Qpx	0.29	£ 4.96			
QQx	0.31	£ 6.15			
QpQ	0.33	£ 7.17			
QQQ	0.36	£ 8.09			
QQq	0.36	£ 7.59			
Qpq	0.42	£ 6.71	0.19	£ 3.10	£15.99 per response

**Figure 3:**
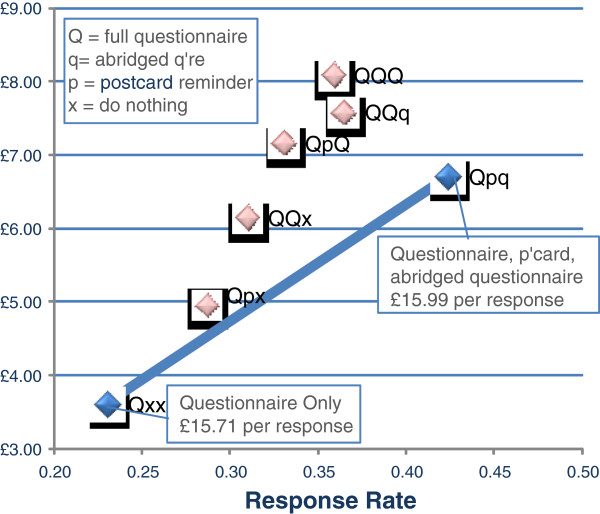
Cost-effectiveness of increasing sample size with different options.

Three options (QpQ, QQq, QQQ) provide a lower response rate at a higher cost than postcard and abridged questionnaire reminders (see Figure 3). These are clearly undesirable. Finally, whilst sending either a postcard or a second questionnaire as a reminder to non-responders (Qpx, QQx) increases response relative to a questionnaire only, it does so inefficiently. Sending out a postcard and then an abridged questionnaire to a subset of the original sample allows us to reach any point on the grey shaded line in Figure 3. As some of these points can provide a higher response rate at a cheaper cost than Qpx and QQx, they also appear to be undesirable in this context.

## Discussion

We have shown that reduced burden (an abridged questionnaire containing fewer questions) following a postcard reminder was the most effective intervention strategy to increase GP response rates to a postal questionnaire. Whilst reduced burden increased GP response rates, this finding did not apply to dentist response rates in our study. We also found that a £20 book voucher incentive did not increase dentist response rates.

Our RCTs had a number of limitations. Our studies did not assess other factors that could predict improved response (e.g. age & gender). We made an assumption that a book voucher mailed with the first questionnaire would appeal to dentists. This incentive may have been insufficient motivation to respond. Monetary incentives have been found to be more effective than non-monetary incentives[3] however this was not feasible due to University regulations that prevented us from mailing cash incentives. It is difficult to ascertain whether a book voucher is viewed as a monetary or non-monetary incentive. Immediate incentives given at the time of the questionnaire have been shown to be more effective than those given on condition of a response [3]. We are unable to determine if the value of the book voucher impacted on our response rate.

Our results found that an incentive led to a reasonable (9%) increase in response rate. The sample size in RCT A was chosen to achieve a 50% response rate for the original PRIME study. If our original response rate had been higher we may not have chosen a 22% difference as the minimum clinically important difference. The fact that it did not reach conventional statistical significance might have been a power issue.

As indicated above, some caution should be used when applying the response rates in RCT B, as the analysis attributes responses to the last prompt given – even if that prompt was not necessarily pivotal in producing the response (not the number of contacts). If this is not the case, then the cost-effectiveness results may be biased against less complex strategies. The cost-effectiveness element of our results also hinges on the implicit assumption that all responses (including those who opt out) have the same value. If this is the case, then those strategies including responses from an abridged questionnaire will tend to be more cost-effective than those with longer questionnaires, given the lower material and data entry costs. Responses from long questionnaires will be more cost-effective in analyses focussing on cost per item of information, and may be more cost-effective if the quality of information can be assessed. As such, the strategy of sending out additional (long) questionnaires to a larger sample may be even more efficient to increase the absolute number of responses than suggested here. Whilst it may increase the response rate it may not provide assurance of a representative or valid response.

Our final response rates to RCT A and RCT B were not high. Following the report by Cummings et al. that up to 1995, response rates of postal questionnaires of healthcare professionals remained constant at approximately 60% [6], Cook et al. demonstrated that by 2005 response rates in surveys of healthcare professionals had slightly declined to an average of 57.5% [4]. Kaner et al. reported doctors describing day to day work pressures and lack of perceived salience as reasons for not completing surveys [15]. Our full questionnaires operationalised multiple theoretical models that resulted in long questionnaires asking seemingly repetitive questions. Additionally, our request to access radiograph and prescribing data may have deterred a larger group from completing a questionnaire.

Interventions to increase response rates may also incur negative consequences. They may lead to differential rates of response or non-response from specific subgroups. In these PRIME surveys [9,10] we received the required pre-specified number of responses from a population sample who had a range of behavior, behavioral simulation and intention, and who reported a range of cognitions. It was not possible to explore the representativeness of responders to these interventions hence there may be a risk of bias. It is also possible that the quality of responses received may differ across the intervention groups but we did not explore quality of response. Further research is needed to explore the effectiveness and impact of other methods to maximise response rates of health care professional postal questionnaires.

## Conclusions

When expecting or facing a low response rate, researchers should carefully identify the most efficient way to boost their response rate. In these RCTs, an abridged questionnaire containing fewer questions following a postcard reminder was the only cost-effective strategy. Although increasing the sampling frame size may be more cost-effective at gaining responses than interventions to increase the number of completed questionnaires within the same sampling frame, this may not strengthen the validity and generalisability of the survey findings.

## Competing interests

The authors declare that they have no competing interests.

## Authors' contributions

LG, RT, GM, DB, MJ, MPE conceived the study. LG, RT, DB, MJ, MPE contributed to the daily running of the study. All authors oversaw the analysis which was conducted by GM and RE. All authors commented on sequential drafts of the paper and approved the final manuscript. Dr Anne Walker conceived the original idea for the PRIME studies.

## Pre-publication history

The pre-publication history for this paper can be accessed here:

http://www.biomedcentral.com/1472-6963/12/250/prepub
